# Gadolinium-Doped Carbon Nanodots as Potential Anticancer Tools for Multimodal Image-Guided Photothermal Therapy and Tumor Monitoring

**DOI:** 10.1021/acsanm.3c03583

**Published:** 2023-09-05

**Authors:** Nicolò Mauro, Roberta Cillari, Cesare Gagliardo, Mara Andrea Utzeri, Maurizio Marrale, Gennara Cavallaro

**Affiliations:** † Laboratory of Biocompatible Polymers, Department of “Scienze e Tecnologie Biologiche, Chimiche e Farmaceutiche” (STEBICEF), University of Palermo, Via Archirafi, 32, 90123 Palermo, Italy; ‡ Department of Biomedicine, Neuroscience and Advanced Diagnostics, University of Palermo, Via del Vespro 129, 90123 Palermo, Italy; § Department of Physics and Chemistry “Emilio Segrè”, University of Palermo, Viale delle Scienze Ed. 18, 90128 Palermo, Italy; ∥ National Institute for Nuclear Physics (INFN), Catania Division, Via Santa Sofia 64, 95123 Catania, Italy; ⊥ Advanced Technology Environment Network Center, Viale Delle Scienze Ed. 18, 90128 Palermo, Italy

**Keywords:** gadolinium, multimodal imaging, photothermal therapy, carbon nanodots, tumor microenvironment, magnetic resonance imaging

## Abstract

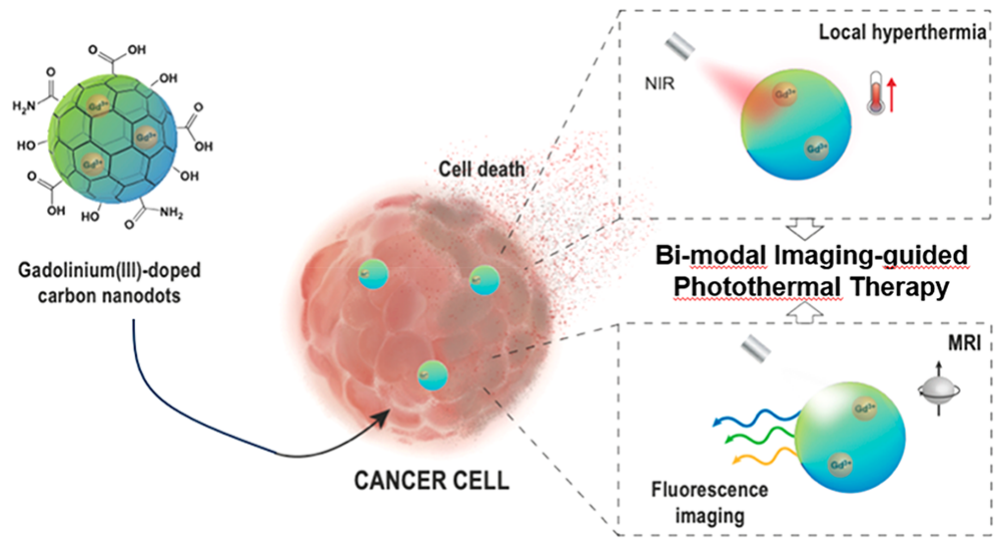

This study focuses on the synthesis and characterization of gadolinium-doped carbon nanodots (CDs-Gd) and their potential applications in multimodal imaging and precision cancer therapy. CDs-Gd were synthesized through a solvothermal decomposition method combining citric acid, GdCl_3_, and urea. The incorporation of Gd^3+^ ions within the carbonaceous structure resulted in stable CDs-Gd with a peculiar architecture that retained optical and paramagnetic properties. Combined characterization techniques confirmed the presence of pH-sensitive COOH functions on the CDs-Gd surface along with the unique lattice structure induced by Gd^3+^ doping. The optical properties of CDs-Gd exhibited a tunable emission spectrum displaying blue-green emission with pH-dependent behavior. Additionally, CDs-Gd exhibited contrast-enhancing properties in *T*_1_-weighted magnetic resonance imaging (MRI) experiments. MRI acquisitions at different Gd^3+^ concentrations and pH values demonstrated the potential of CDs-Gd as contrast agents for monitoring pH changes in an aqueous environment. We found that the relaxivity of CDs-Gd at pH 5.5 (tumor, 11.3 mM^–1^ s^–1^) is roughly 3-fold higher than that observed at pH 7.4 (physiological, 5.0 mM^–1^ s^–1^) and outperformed clinical standards such as γ-butyrol (3.3 mM^–1^ s^–1^). Monitoring pH changes in tumor microenvironment (TME) is crucial for evaluating the effectiveness of anticancer treatments and understanding tumor progression. Furthermore, CDs-Gd demonstrated concentration-dependent photothermal conversion ability in the near-infrared (NIR) region, allowing for efficient heat generation under laser irradiation. This indicates the potential application of CDs-Gd in image-guided photothermal therapy (IG-PTT) for cancer treatment. The in vitro studies on MCF-7 (breast cancer) and 16-HBE (healthy bronchial epithelium) cell lines demonstrated that CDs-Gd exhibited high biocompatibility (cell viability >80%). However, upon NIR activation, they showed potent anticancer effects by inhibiting tumor cell proliferation and inducing apoptosis selectively in cancer cells. In conclusion, the synthesized CDs-Gd nanoparticles possess unique optical, photothermal, and MRI contrast properties, making them promising candidates for multimodal imaging-guided precision cancer therapy applications.

## 1 Introduction

Despite significant advancements in cancer research, resulting in improved survival rates and reduced mortality, currently available anticancer treatments such as chemotherapy and radiation therapy are nonspecific and often lead to severe side effects that negatively impact patients’ quality of life.^1,2^ Thus, there is an urgent need to develop personalized anticancer approaches that are specific and minimally invasive, overcoming drawbacks of traditional therapies.^3^ The emergent field of cancer theranostics fits in this contest by providing innovative therapeutic tools for performing targeted anticancer treatments guided by bioimaging techniques.^4^ By integrating both diagnostic and therapeutic elements within a single nanostructure,^5^ theranostic platforms aim to implement targeted therapeutic interventions while simultaneously monitoring both drug localization and the therapeutic outcome in real time.^6,7^ For instance, theranostic nanoplatforms with pH-sensitive contrast properties can be exploited to monitor tumor microenvironment (TME) changes during the regression of a cancer disease. Thus, the efficiency of treatments can be optimized patient-to-patient as a function of the observed efficacy. To achieve this, carbon nanodots (CDs) have attracted great interest because of their intrinsic environment-sensitive fluorescence imaging contrast properties and strong photothermal conversion efficacy, accompanied by many additional benefits, including low cost, ease of synthesis, high water solubility, and biocompatibility.^6,8^ The inherent photoluminescence of CDs makes them excellent probes for fluorescence imaging (FLI)—a worthwhile technique that has been widely used in early disease diagnosis and therapeutic efficiency evaluation because of its high sensibility and temporal resolution.^9^ For fluorescence-imaging applications, CDs are superior to both fluorescent organic dyes and nanoparticles having similar emission properties, such as semiconductor quantum dots, in terms of chemical and photostability, high biocompatibility, and tunable optical properties.^10^ CDs can be used for labeling cancer cells, allowing their detection by flow cytometry and their investigation at the molecular level by fluorescence microscopy, enabling a comprehensive understanding of cancer intracellular processes and an accurate evaluation of tumor progression and treatment response.^11,12^ Moreover, CDs are also effective therapeutic tools as their ability to convert absorbed light into heat can be exploited to directly damage cancer cells and stimulate immune cells by inducing a local hyperthermia (42–49 °C).^13−16^ Near-infrared (NIR)-based photothermal therapy (PTT) also offers a high selectivity for cancerous tissues due to the spatiotemporal control of external light sources which can be specifically directed to the diseased lesion on-demand.^17^ Due to its simplicity, noninvasiveness, and remote-controlled features, imaging-guided photothermal therapy (IG-PTT) represents a very effective and clever evolution of PTT to achieve highly specific treatments with negligible off-target effects.^18,19^

However, limitations of FLI methods still hamper theranostic applications of CDs in IG-PTT, which remains challenging, especially for the treatment of deep tissues. In fact, light absorption and scattering phenomena occurring in biological tissues limit the tissue penetration depth of light radiation, affecting the spatial resolution of the output imaging signal.^20^ To effectively enable the in vivo monitoring of CDs, they should be engineered with contrast agents resulting in multimodal imaging nanosystems that benefit from the combination of complementary imaging modes (i.e., magnetic resonance imaging-MRI and FLI).^21,22^ The association of FLI and MRI is very interesting, as MRI can address the drawbacks of FLI application in vivo by providing deep-penetration imaging with high soft-tissue contrast and superior spatial resolution. However, the most widely used MRI contrast agents are based on gadolinium(III) complexes, which could release free ions displaying marked toxicity in vivo.^23,24^ Although these compounds are safer than free Gd^3+^ ions, their use still implies significant risks related to their dissociation in human serum and their body accumulation.^25^ The incorporation of Gd^3+^ within nanomedicines could improve their stability and consequently their safety profile.^26,27^ Likewise, multimodal imaging nanosystems combining the advantages of FLI and MRI can be obtained by introducing Gd^3+^ ions into nanoparticles already presenting intrinsic photoluminescence. Thereby, some report proposed gadolinium-functionalized gold nanomaterials, quantum dots, or carbon nanomaterials for efficient FLI/MRI bimodal imaging.^28,29^ However, these nanosystems still rely on gadolinium coordination on the nanoparticle’s surface, which could cause toxicity due to Gd^3+^ dissociation and release after administration. Moreover, they do not display pH-sensitive MRI contrast features, thereby precluding TME sensing and monitoring applications. Herein, we developed a solvothermal synthesis of gadolinium-doped carbon nanodots, namely, CDs-Gd, endowed with narrow size distribution, high NIR photothermal conversion, and high amount of surface carboxyl functions. The proposed gadolinium-doped CDs offer a unique advantage over other nanosystems, as they incorporate Gd^3+^ ions within structural defects of the CDs’ core. This design avoids potential toxicity from Gd^3+^ dissociation and provides high pH sensitivity owing to free carboxyl groups on the CDs’ surface. Besides, presenting both the paramagnetic properties of gadolinium and the optical properties of CDs, such multifunctional nanosystems could be used to perform efficient pH-dependent MRI/FLI multimodal imaging-guided photothermal therapy, providing huge potential in precision cancer therapy and monitoring.

## 2 Materials and Methods

### 2.1 Materials

Urea (99%), citric acid (99.5%), *N*,*N*-dimethylformamide (DMF), gadolinium chloride (GdCl_3_), xylenol orange tetrasodium salt (XO), Sephadex G-15 and G-25, and Dulbecco’s phosphate buffered saline (DPBS) were purchased from Merck Life Science S.r.l. (Milan, Italy).

Dulbecco’s modified Eagle’s medium (DMEM), fetal bovine serum (FBS), l-glutamine, penicillin, streptomycin, and amphotericin B were purchased from EuroClone S.p.A. (Milan, Italy). 4′,6-Diamidino-2-phenylindole (DAPI) was purchased from Life Technologies. The CellTiter 96 AQueous One Solution Cell Proliferation Assay (MTS) was purchased from Promega (Milan, Italy).

Human breast cancer cell line MCF-7 was obtained from “Istituto Zooprofilattico Sperimentale della Lombardia e dell’ Emilia Romagna”, Italy. Human bronchial epithelial cells 16-HBE were purchased from Life Technologies (ThermoFisher Scientific). Cells were cultured in 75 cm^2^ culture flasks with a vented filter cap (Biosigma) at 37 °C in a humidified atmosphere of 5% CO_2_. DMEM used for cell culture was supplemented with 10% (v/v) FBS, 100 units/mL penicillin G, 100 μg mL^–1^ streptomycin, 0.1% (v/v) amphotericin B, and 2 mM l-glutamine.

### 2.2 Synthesis of Gadolinium-Doped Carbon Nanodots (CDs-Gd)

CDs-Gd were prepared from urea (6 g, 99.9 mmol), citric acid (3 g, 15.6 mmol), and gadolinium(III) chloride hexahydrate (579.8 mg, 1.56 mmol) in anhydrous DMF. The mixture was kept under solvothermal conditions at 160 °C in a steel autoclave (Büchi AG, Miniclave steel type 3, Gschwaderstrasse, Switzerland) for 4 h. Then, the organic solvent was removed by rotary evaporation (3 mbar, 45 °C), and the crude was suspended in ultrapure water by sonicating (15 min × 3). The obtained dispersion was filtered through a paper filter and, subsequently, purified by size exclusion chromatography (SEC) using water as eluant and a glass column packed with, in turn, Sephadex G-15 and G-25 as stationary phase. The collected fractions were gathered based on their UV/vis absorption spectra, and the most interesting, named CDs-Gd, was selected for deeper characterization. Finally, the pH of the selected fraction was adjusted to 1.0, and the product was further purified by SEC using water as eluant and Sephadex G-10 as stationary phase. The yield of CDs-Gd was 109.3 mg.

### 2.3 Physicochemical Characterization of Gadolinium-Doped Carbon Nanodots (CDs-Gd)

Fourier-transform infrared spectra (FT-IR) were recorded using a PerkinElmer Spectrum Two IR spectrometer (Waltham, MA) in the range 4000–400 cm^–1^. Samples were prepared as KBr pellets and dried under vacuum before performing the analysis.

^1^H NMR spectra were recorded by using a 400 MHz Advance II 400 spectrometer (Bruker Biospin).

X-ray photoelectron spectroscopy (XPS) spectra were collected with a PHI 5000 VersaProbe II system (ULVAC-PHI, Inc.) equipped with a Kα source, 1486.6 eV. The sample was prepared by depositing 500 μL of an aqueous solution of CDs-Gd (0.5 mg mL^–1^) on an aluminum plate, which was dried under vacuum for 24 h before performing the analysis. Acquisition parameters: a beam of 100 μm ϕ, 25 W; time per step: 10 ms; energy step: 0.05 eV; pass energy: 23.50 eV; analyzer mode: FAT. The carbon (C 1s) line at 284.8 eV was used as reference energy.

The high-resolution transmission electron microscopy (HR-TEM) was performed on a JEM-2010 (JEOL) microscope at 200 kV electron energy. The sample was prepared by depositing a drop of an aqueous solution of CDs-Gd on a commercial 400 μm mesh Cu-grid (Plano 01824) covered by a holey amorphous carbon film with a nominal thickness of 3 nm.

Atomic force microscopy (AFM) micrographs were obtained on a FAST-SCAN microscope equipped with a closed-loop scanner (*X*, *Y*, and *Z* maximum scan region: 35, 35, and 3 μm, respectively). The analysis was performed in soft tapping mode using a FAST-SCAN-A probe with an apical radius of 5 nm operating at 1400 kHz (*k*: 18 N/m). The sample was prepared by depositing 15 μL of a 25 μg mL^–1^ aqueous solution of CDs-Gd on MICA substrates which was dried under vacuum before performing the analysis.

Differential scanning calorimetry (DSC) and thermogravimetric analysis (TGA) of the CDs-Gd sample were performed with a DSC/TGA 131 EVO instrument (by SETARAM Instruments). Measurements were performed under a nitrogen atmosphere (flow rate 1 mL min^–1^) in a closed alumina crucible. Data were recorded within the range 30–600 °C using a heating rate of 10 °C min^–1^.

### 2.4 Determination of Gd^3+^ Ions in Gadolinium-Doped Carbon Nanodots (CDs-Gd)

The amount of Gd^3+^ ions in CDs-Gd was evaluated spectroscopically using xylenol orange tetrasodium salt (XO) as metal indicator.^30^ A sample of CDs-Gd (2 mg), both before and after purification at pH 1, was mineralized by in turn treating it with 10% v/v HNO_3_ for 5 min at 100 °C and 15 min at 200 °C using a microwave synthesizer Discover SP (CEM). At the end of the process, the sample was cooled, and the pH was adjusted to 5.8. A solution of XO in acetate buffer pH 5.8 (16 μg mL^–1^; 950 μL) was added to 50 μL of each sample. Then, the absorption spectrum was recorded within the range 400–600 nm and the amount of Gd^3+^ was calculated by comparing the 573 nm/433 nm absorbance ratio with a calibration curve obtained with GdCl_3_ standards (10–30 μM) in acetate buffer solution pH 5.8 (*R*^2^ = 0.993). The Gd^3+^ loading was expressed as the weight percentage of Gd atoms entrapped in 100 mg of CDs.

### 2.5 Optical Characterization

The UV/vis absorption spectrum of CDs-Gd was recorded using a double-beam spectrophotometer (Shimadzu UV-2401PC) within the range 200–800 nm (1 nm bandwidth). CDs-Gd spectra were recorded in either water, 5 mM acetate buffer (pH 5.5, 5 mM phosphate buffer (pH 6.4,), or 5 mM phosphate buffer (pH 7.4).

The emission spectra of CDs-Gd aqueous solutions were recorded using a Jasco FP-8500 spectrofluorometer within the range 360–750 nm (λ_ex_ = 350 nm) and 460–750 nm (λ_ex_ = 450 nm). 3D emission spectra of CDs-Gd solutions in water, 5 mM acetate buffer (pH 5.5), 5 mM phosphate buffer (pH 6.4), or 5 mM phosphate buffer (pH 7.4) were obtained using the same experimental set (λ_ex_ = 350–600 nm, λ_em_ = 360–750 nm).

The photothermal kinetics were evaluated following the sample temperature of CDs-Gd aqueous solutions (from 0.1 to 0.5 mg mL^–1^) over the irradiation time and using an 810 nm diode laser (GBox 15A/B GIGAA laser) at 2.5 W cm^–2^ for 300 s. At scheduled time intervals (Δ*t* = 50 s), the sample temperature was measured by using an infrared optical fiber (CEM). The photothermal kinetic obtained using ultrapure water was used as control.

### 2.6 Magnetic Resonance Imaging (MRI)

In vitro MRI acquisitions were performed using a Philips Achieva 1.5T MRI clinical scanner equipped with an eight-channel phased-array-head coil. The samples consisted of 3 mL of CDs-Gd solutions (0.01–0.5 mg mL^–1^, equivalent to 1.1–10.8 μM of Gd^3+^) prepared in acetate buffer (pH 5.5, 5 mM), phosphate buffer (pH 6.4, 5 mM), or phosphate buffer (pH 7.4, 5 mM). The samples were placed in vials, which were then inserted into a nonmagnetic holder and positioned inside the head coil to ensure a good signal-to-noise ratio.

Measurements of the longitudinal relaxation time *T*_1_ were performed by using an inversion recovery sequence. The acquisition parameters included an echo time (TE) of 15 ms, a maximum repetition time (TR) of 4000 ms, matrix size of 256 × 256, slice thickness of 2.5 mm, and a field of view of 25.6 × 25.6 cm^2^. Images were acquired with variable inversion times (TI) ranging from 25 to 3000 ms.

To analyze the data, Python code was developed to identify the region of interest (ROI) at the center of each sample and calculate the average signal intensity as well as its standard deviation. *T*_1_ values were obtained by fitting the acquired data to the equation *M*(TI) = *M*_0_ × (1 – *b* × e^–TI/*T*_1_^), where *M*_0_, *b*, and *T*_1_ are the fitting parameters. Finally, the relaxation rate (*R*_1_) values were determined to be the reciprocal of *T*_1_ (s^–1^).

### 2.7 Biological Characterization of the Gadolinium-Doped Carbon Nanodots (CDs-Gd)

Cell uptake studies of CDs-Gd were performed by fluorescence microscopy on MCF-7 and 16-HBE cell lines. For each experimental set, cells were seeded on 8-well Nunc Lab-Tek chambered coverglass (Thermo Fisher Scientific) at a 10^4^ cell density and cultured in complete DMEM. After 24 h, the culture medium was withdrawn, and cells were incubated with a CDs-Gd dispersion in complete DMEM (250 μL, 0.15 mg mL^–1^). At scheduled time intervals (2, 6, and 24 h), the medium was replaced with DPBS pH = 7.4, and cells were washed three times. Cells were fixed using 4% buffered formalin and treated with DAPI (50 μL × 5 min) to stain nuclei. Cell uptake micrographs were obtained with a fluorescence microscope Axio Cam MRm (Zeiss) (100×).

The cytocompatibility profile of CDs-Gd was assessed in vitro on both breast cancer cell (MCF-7) and human bronchial epithelial cell (16-HBE) lines using the MTS assay (Promega). Briefly, cells were seeded in a 96-well plate at 1.5 × 10^4^ cells/well density and cultured for 24 h. Then, the culture medium was replaced with either CDs-Gd dispersions in DMEM (200 μL, 0.05–0.3 mg mL^–1^) or 200 μL of fresh medium (negative controls). After 24 or 48 h of incubation, the medium was removed and each well was washed twice with DPBS (pH 7.4) before incubating with a MTS solution (120 μL, 1:5 MTS assay solution/DMEM) for 2 h. Finally, cell viability was calculated measuring the absorbance at 492 nm using a microplate reader (Multiskan, Thermo, UK) and comparing each value with those obtained for the negative control (100% cell viability).

The near-infrared (NIR)-induced photothermal effect was assessed on the same cell lines considered for the cytocompatibility study and using the MTS assay. In particular, cells were treated as previously described, but they were further irradiated with a pulsed 810 nm diode laser (GBox 15A/B diode GIGAA laser) with a power density of 5 W cm^–2^ for 300 s before performing the MTS assay.

For the hemocompatibility assay, a 4% v/v suspension of erythrocytes in DPBS pH 7.4 (200 μL), isolated from a whole blood sample by centrifugation (500*g* × 10 min × 2), was treated with increasing amount of CDs-Gd (0.01–0.5 mg mL^–1^) for 1 h. After that, each sample was centrifuged (500*g* × 5 min), and the absorbance at 520 nm of the supernatant was measured by using a microplate reader (Multiskan, Thermo, UK). The number of erythrocytes disrupted was estimated considering the absorbance obtained incubating Triton X-100 and considering it as 100% of lysis.

All experiments were performed in triplicate, and results are expressed as mean values.

### 2.8 Statistical Data Analysis

Statistical analysis was performed by the two-tailed *t*-test of the data analysis package from Origin Lab. Comparisons were considered statistically significant at *p* < 0.05 (∗), *p* < 0.01 (∗∗), and *p* < 0.001 (∗∗∗).

## 3 Results and Discussion

### 3.1 Synthesis of Gadolinium-Doped Carbon Nanodots (CDs-Gd)

**Figure 1:**
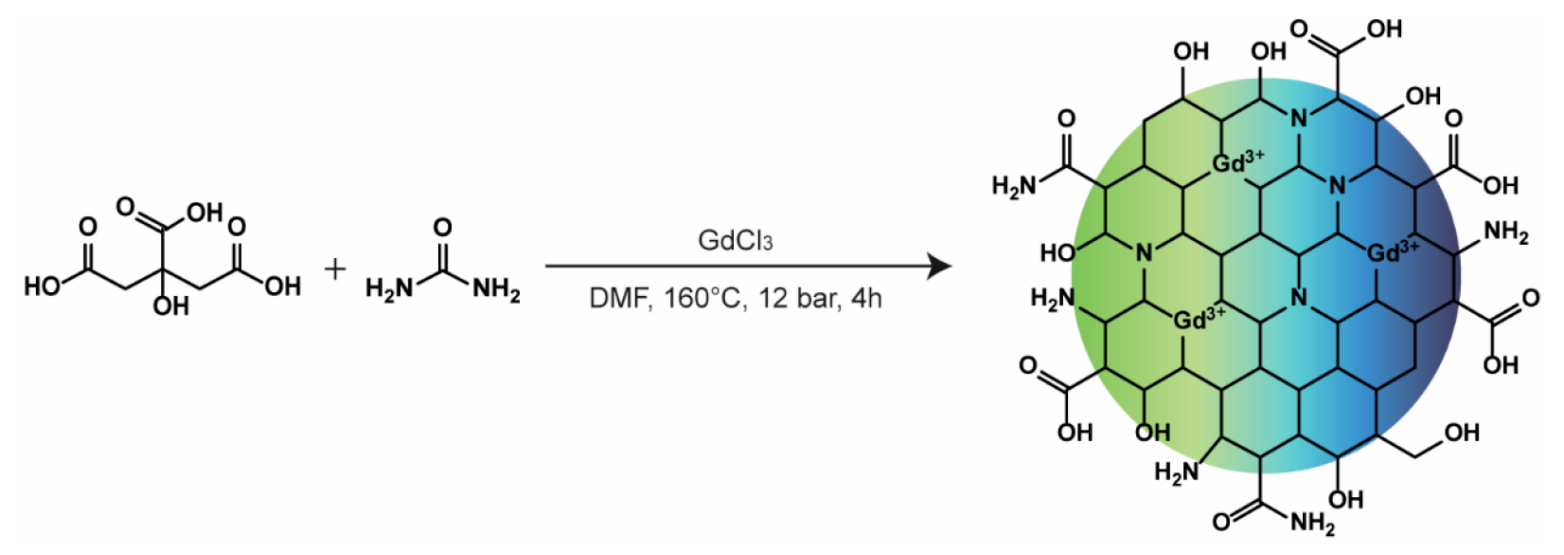
Schematic representation of the synthetic pathway employed for the synthesis of CDs-Gd.

Gadolinium-doped carbon nanodots (CDs-Gd) were obtained by a one-pot protocol consisting of the solvothermal decomposition of citric acid, GdCl_3_, and urea in DMF (Figure 1). The solvothermal decomposition of citric acid and urea is a widely explored and very effective technique to easily obtain fluorescent N-doped carbon nanodots (CDs) with high reaction yield and endowed with good NIR photothermal conversion ability as well as suitable fluorescence quantum yield (QY > 30%) helpful in IG-PTT.^31−33^ The rationale adopted relies on the introduction of GdCl_3_ among the molecular precursors used during the bottom-up synthesis process, which would provide Gd^3+^ ions that can be entrapped in the carbonaceous structure, thus leading to CDs with structural defects holding both optical and paramagnetic properties, useful for multimodal FLI/MRI imaging application. The amount of GdCl_3_ was chosen by considering that a 10:1 molar ratio of citric acid to Gd^3+^ provides the formation of a stable complex, where two molecules of citric acid bind one Gd^3+^ ion. This leads to a mixture that contains both the 2:1 citric acid/Gd^3+^ complex (3.12 mmol) and free citric acid molecules (12.48 mmol), which can participate in Gd^3+^ doping and the formation of crystalline nitrogen-doped CDs during the solvothermal process, respectively. A higher amount of doping agent would increase structural defects in carbonaceous core, significantly reducing the size of the CDs. During the solvothermal decomposition, the reaction developed a pressure of 12 bar which typically give rise to crystalline green-emitting CDs of 1–5 nm in diameter.^33^ Thus, after the solvothermal decomposition process, the organic solvent was removed by rotary evaporation and the crude was redispersed in ultrapure water to be purified by SEC using a Sephadex stationary phase packed with an increasing cutoff (G10–G15–G25) in order to separate fractions with different size distributions.^34^

In particular, a fraction showed a complex and interesting absorption spectrum with green emission, whereas the others displayed a sharp emission peak in the blue region (340 nm) and a poorly defined absorption spectrum, probably due to high inhomogeneity (Figure S1). Therefore, only a fraction, henceforth named CDs-Gd, was chosen as the most promising and was selected for further characterizations. To remove the Gd^3+^ ions eventually complexed on the CDs-Gd surface, we further purified the selected fraction by size-exclusion chromatography (SEC) at pH 1. Under these conditions, any Gd^3+^ ions eventually complexed by carboxyl groups are released due to the protonation of the COO^–^ groups. Thus, unlike other hybrid nanosystems reported in the literature, where Gd^3+^ is complexed on the surface and thus free to diffuse in physiological fluids, the prepared CDs-Gd sample was characterized by stable Gd^3+^–CDs bonds and the absence of ion release over time under both acidic and physiological conditions (Figure S2).

### 3.2 Chemical, Structural, and Morphological Characterization of Gadolinium-Doped Carbon Nanodots (CDs-Gd)

The amount of Gd^3+^ integrated in CDs-Gd was evaluated spectrophotometrically employing xylenol orange tetrasodium salt (XO) as metal indicator.^30^ Briefly, XO is an organic dye having two main absorption peaks in the visible region at 433 and 573 nm (Figure S3). In the presence of metal cations, such as Gd^3+^, the relative intensities of the two absorption bands change as a consequence of ion complexation.^35^ Before the spectrophotometric assay was performed, the sample of CDs-Gd was subjected to a mineralization process by dissolution in HNO_3_ 10% and high-temperature treatment, in order to release the Gd^3+^ ions entrapped in the nanoparticle core, by degrading the carbonaceous structure. Comparing the 573 nm/433 nm absorbance ratio with a calibration curve obtained with GdCl_3_ standards, it was calculated that Gd^3+^ ions represent the 0.34% w/w of CDs-Gd (Figure S3). This amount of incorporated gadolinium results to be among the highest reported in the literature.^36^ We conducted a similar analysis on a purified CDs-Gd sample, which had undergone size-exclusion chromatography (SEC) at pH 1 to remove any Gd^3+^ ions that may have been complexed by the COO– carboxyl groups on the surface of the nanomaterial. We found that the amount of Gd^3+^ ions in the purified sample is about 0.76% w/w, indicating that Gd^3+^ is mostly enclosed within the core of the synthesized nanostructures.

**Figure 2:**
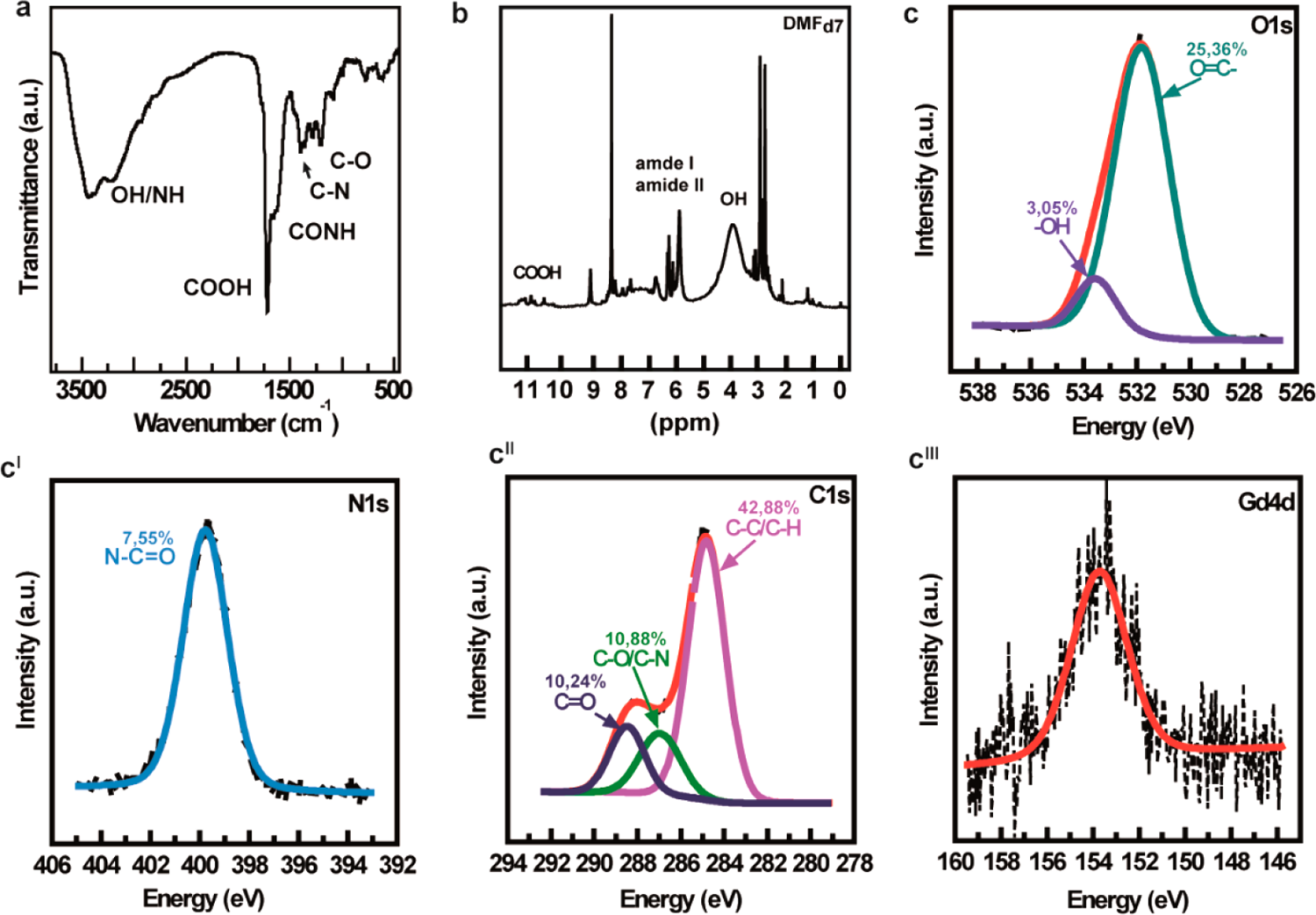
Chemical characterization of CDs-Gd. (a) FT-IR spectrum of CDs-Gd prepared as a KBr pellet. (b) ^1^H NMR spectrum of CDs-Gd solution in DMF-*d*_7_. (c–c^III^) XPS analysis and deconvolutions of the rough spectra.

The chemical composition of CDs-Gd was investigated by combining different analysis techniques such as FT-IR, ^1^H NMR, and XPS. The FT-IR spectrum of CDs-Gd (Figure 2a) presents the typical bands of C=O asymmetric stretching, attributable to carboxyl (1715 cm^–1^) and amide I (1665 cm^–1^) groups, OH and NH stretching (3400 and 3200 cm^–1^), C–N (1385 cm^–1^) stretching, and N–C–O (1200 cm^–1^) vibrations. These diagnostic bands suggest that the surface of CDs-Gd is rich in polar functional groups, including hydroxyl, amine, amide, and carboxyl groups, which are desirable for easier manipulation and processing of the product, as they confer high dispersibility in different solvents and can be easily exploited for further surface functionalization.^6,37^ The FT-IR data were confirmed by the ^1^H NMR spectrum (Figure 2b) which shows resonances related to carboxyl (10.0–10.2 ppm), amide (5.69–7.92 ppm), and hydroxyl (3.84 ppm) groups. Besides, XPS analysis also provided evidence of the presence of carboxyl, amide, and hydroxyl groups, together with Gd^3+^ ions. In particular, Figure 2c shows the typical peaks of hydroxyl and carbonyl groups in the O 1s signal (532 eV), with a major contribution of O=C (25.36%). Considering that only amide groups contribute to the N 1s signal (∼400 eV), with an abundance of 7.55% (Figure 2c^I^), it can be estimated that most of the carbonyl groups are O=C–O^–^. The C 1s signals (Figure 2c^II^) support the interpretation mentioned above, as all the characteristic contributions of C–C/C–H (∼285.5 eV), C–O/C–N (∼287 eV), and O=C (∼288.5 eV) can be clearly distinguished. In agreement with the spectrophotometric data reported above, the low intensity of the Gd 4d signal at 153.8 eV (Figure 2c^III^) suggests that Gd^3+^ ions are present only in small amounts (<1%) in the CDs-Gd sample. Besides, in XPS, Gd signals appear at binding energies that depend on the oxidation state od ions present in the sample. As a rule, the Gd 4d signal can be distinguished in the range 140–160 eV, and the specific binding energies for paramagnetic Gd^3+^ species are commonly observed at around 153–154 eV. The prevalent binding energy observed in the CDs-Gd sample is at 154 eV, suggesting that gadolinium is present as Gd^3+^ ions.

**Figure 3:**
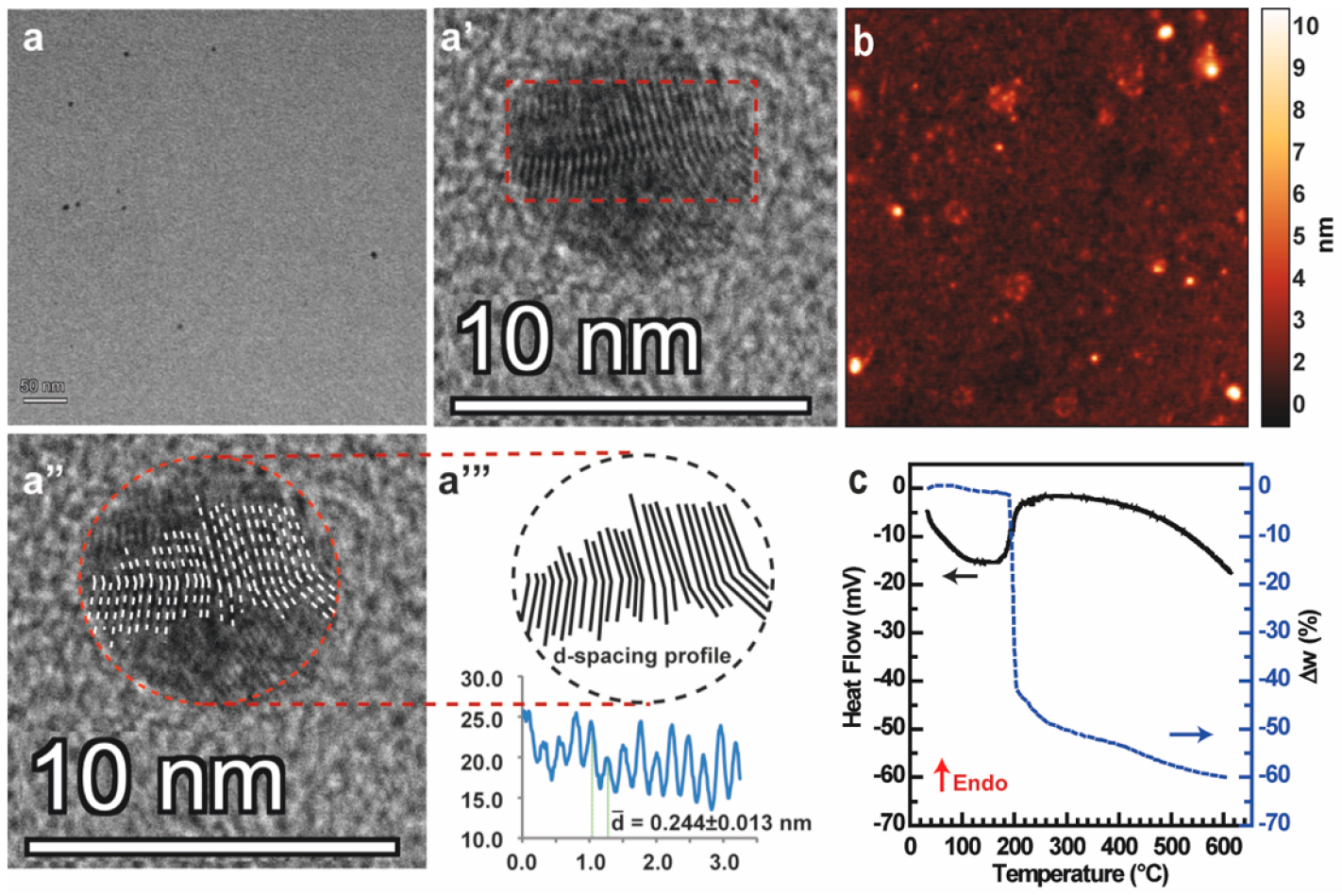
Structural and chemical characterization of CDs-Gd. (a–a^II^) HR-TEM micrographs and (a^III^) details of the *d*-spacing profile. (b) AFM micrograph. (c) DSC (black) and TGA (blue) thermograms.

Assuming the presence of Gd^3+^ within the nanosystem, we decided to investigate the structure of the carbon core in detail via high-resolution transmission electron microscopy (HR-TEM). HR-TEM micrographs displayed homogeneous crystalline nanostructures of about 5 nm (Figure 3a–3a^I^), presenting a very interesting *d*-spacing pattern. The core of the crystal is characterized by an unconventional lattice fringe in which the lattice planes are not aligned in parallel throughout the crystal. Instead, they appear to be deflected by some structural defects attributable to Gd^3+^ doping (Figure 3a^I^–3a^II^). Deflections do not affect the *d* spacing, which remains roughly 0.244 ± 0.013 nm (Figure 3a^III^), indicating that the crystal lattice maintains a regular structure attributable to common nitrogen-containing CDs.^38^ To the best of our knowledge, most of the gadolinium-containing carbon dots reported in the literature have either amorphous or regular crystalline cores and carboxyl groups at the surface that coordinate Gd^3+^ ions.^39,40^ Besides, they do not display the peculiar lattice frame here observed, which therefore seems to be a unique feature of these hybrid CDs-Gd nanosystems.^41,42^ It can be assumed that the structural defects observed are caused by the incorporation of Gd^3+^ ions within the crystal lattice. Compared to other CDs reported in which Gd^3+^ ions are chelated by surface carboxyl groups, the prevalent incorporation of Gd^3+^ within the carbonaceous structure provides a huge advantage because ions are retained inside the CDs-Gd hybrids, thus avoiding toxic effects owing to uncontrolled release throughout the body.^43^

The size distribution of CDs-Gd was established by atomic force microscopy (AFM). Micrographs showed in Figure 3b display spherical objects of 5.10 ± 0.20 nm average heights, corroborating the HR-TEM data. The sample appears homogeneous with very few aggregates, demonstrating that the purification process employed was effective.

To assess the thermal stability of CDs-Gd, differential scanning calorimetry (DSC) and thermogravimetric (TGA) analyses were performed within the range 10–600 °C (Figure 3c). The sample was stable up to 180 °C because no significant transitions or weight loss can be appreciated in DSC and TGA thermograms. The DSC thermogram shows a broad endothermic peak from 190 to 600 °C, indicating the sharp decomposition of surface polar groups (i.e., hydroxyl, carboxyl, and amide groups) on the CDs’ surface. This interpretation is supported by the observed weight loss (approximately Δ*w* = −60%) above 190 °C in the TGA thermogram. Besides, the calculated amount of surface functional groups from the decomposition aligns with XPS data, which indicates that they constitute about 61% of the sample.

### 3.3 Optical Properties of Gadolinium-Doped Carbon Nanodots (CDs-Gd)

**Figure 4:**
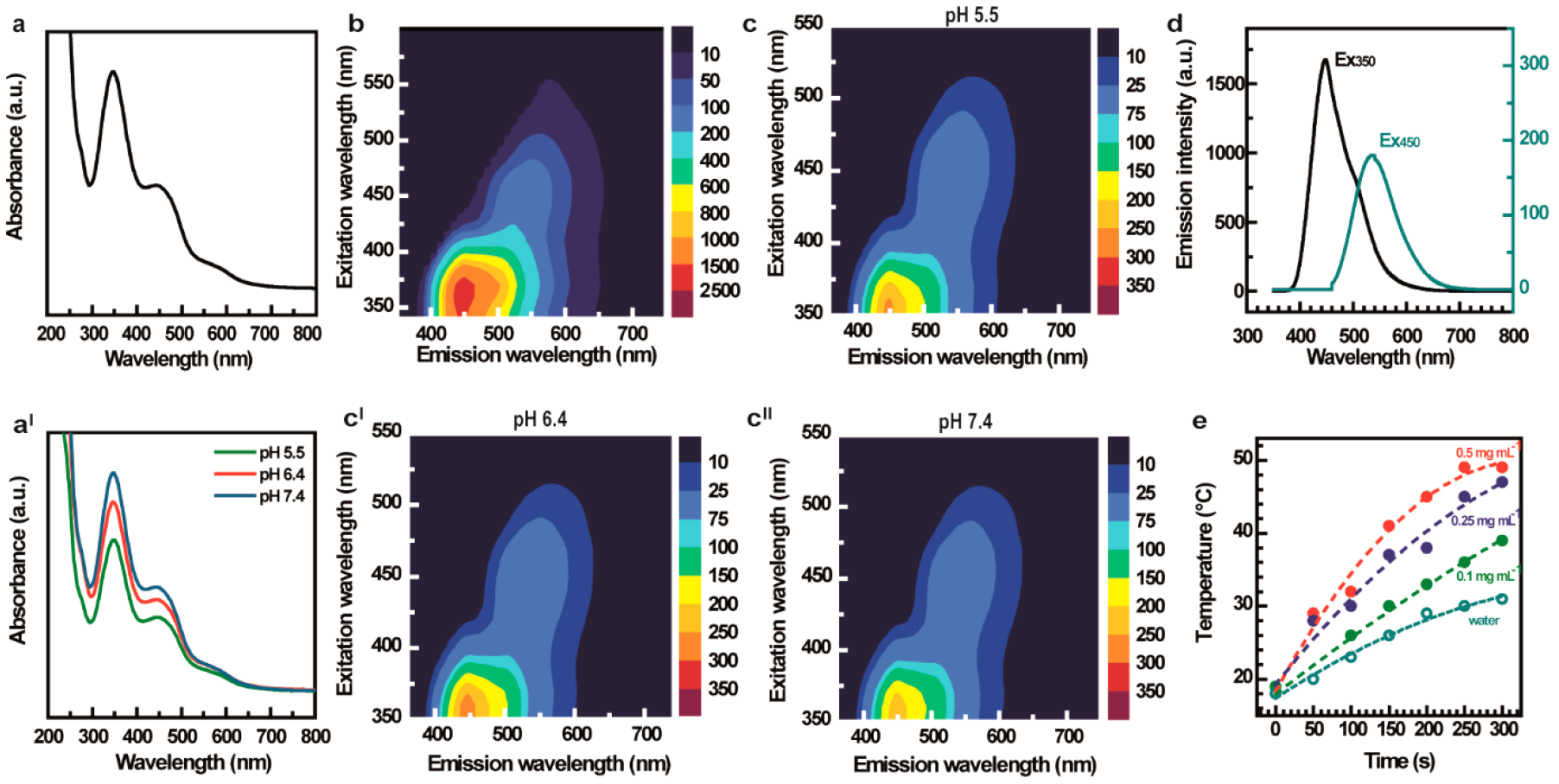
Optical characterization of CDs-Gd. (a) UV–vis absorption spectra of CDs-Gd in water dispersion and in different buffers (a^I^). (b) 3D-fluorescence emission spectrum of CDs-Gd in water. (c–c^II^) 3D-fluorescence emission spectra of CDs-Gd in buffered solutions at pH 5.5, 6.4, and pH 7.4. (d) 2D-emission spectra of CDs-Gd aqueous solutions. (e) Temperature kinetics of CDs-Gd aqueous dispersions or ultrapure water irradiated with an 810 nm diode laser for 300 s (2.5 W cm^–2^).

The UV–vis absorption spectrum of CDs-Gd presents a complex absorption profile, with broad peaks extending throughout the visible region, characterized by three primary absorption peaks at 350 and 450 nm and a shoulder at 575 nm (Figure 4a). These peaks can be attributed to electronic transitions involving surface states that are associated with the diverse nature of functional groups that characterize the surface of CDs-Gd.^44^ It is also noticeable a huge absorption below 300 nm, which is commonly assigned to band-to-band core transitions.^34^ It might be noticed that the optical features of CDs dispersions in water are stable over time as well as under an extensive excitation time (Figure S4). It is noteworthy that the emission intensity does not undergo a significant photobleaching even if the sample is excited for 5 min at 350 nm (the fundamental state is reached after 600 s of recovery time in the dark). To investigate the pH dependence of CDs-Gd’s optical properties, we also recorded absorbance spectra in buffer solutions at pH 5.5, 6.4, and 7.4 (Figure 4a^I^). The goal was to determine the pH sensing capabilities of these nanostructures, which could be exploited to monitor changes in the tumor microenvironment (TME) during anticancer treatments. The selected pH values were chosen to simulate the pH conditions of physiological tissues (pH 7.4), the TME (pH 6.4), and intracellular endosomes (pH 5.5).^45^ As shown in Figure 4a^I^, there is not an appreciable shift of the main absorption peaks, indicating that the shape of the absorption spectra is not affected by pH changes.

The wide absorption spectrum of CDs-Gd corresponds to multicolor fluorescence emission that can be tuned as a function of the excitation wavelength (Figure 4b–d). The emission spectrum indeed displays a main emission band in the blue-green region (QY_blue_ = 11.8%, QY_green_ = 6.1%), as well as a tail that extends to the red region (QY = 2.1%; Figure 4b). The strong fluorescence in the blue/green region represents a very interesting property of CDs-Gd, suggesting its promising application in cancer cell labeling and intracellular fluorescence tracking. Unlike the absorption spectrum, the emission properties of CDs-Gd seem to be influenced by the pH (Figure 4c–c^II^). Specifically, the blue emission is more intense at acidic pH values, offering an interesting advantage to monitor pH changes inside cancer cells and the TME during treatment.

To investigate the photothermal conversion ability of CDs-Gd in the NIR region, we measured the temperature kinetics of CDs-Gd aqueous dispersions (at concentrations ranging from 0.1 to 0.5 mg mL^–1^) over time under irradiation with a laser diode (λ = 810 nm, 5 W cm^–2^). The resulting hyperthermic kinetics are reported in Figure 4e, revealing that exposure of CDs-Gd solutions to moderate-power-density NIR laser light causes a significant temperature increase across all concentrations tested. The photothermal conversion observed for the CDs-Gd solutions can be explained by the absorption tail clearly visible from 700 to 800 nm (Figure 4a). In more detail, compared to ultrapure water (Δ*T* = 13 °C), the temperature increases (Δ*T*) recorded for samples with concentration of 0.5, 0.25, and 0.1 mg mL^–1^ were 31, 29, and 20 °C, respectively. These results indicate that a concentration-dependent light-to-heat conversion occurs in the presence of CDs-Gd. The significant heat generation observed under mild conditions with even small amount of CDs-Gd (e.g., 0.25 mg mL^–1^) and low exposure (300 s) can provide a suitable hyperthermic state (42–49 °C) helpful in cancer PTT.

Overall, the unique optical properties of the CDs core in terms of absorption, emission, and photothermal conversion are not compromised by the presence of gadolinium ions in the core structure. This suggests that CDs-Gd could be a potential photothermal agent for image-guided anticancer treatments (IG-PTT).

### 3.4 Contrast Properties in Magnetic Resonance Imaging (MRI)

**Figure 5:**
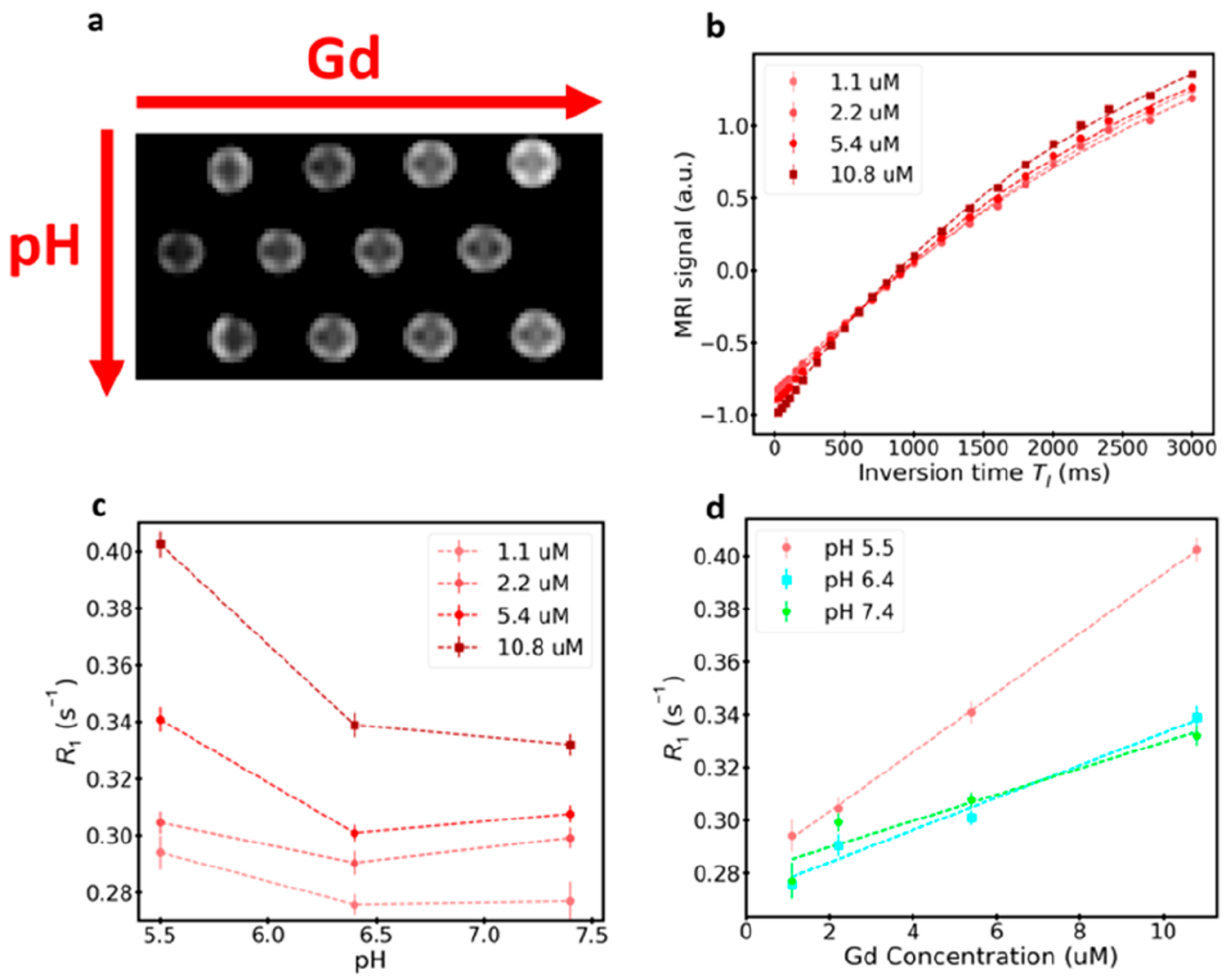
(a) *T*_1_-weighted MR acquisition on CDs-Gd for samples prepared with various Gd^3+^ concentrations (between 1.1 and 10.8 μM) and for various pH values (i.e., 5.5, 6.4, and 7.4) (TR = 4000 ms, TI = 3000 ms). (b) MRI signal as a function of the inversion time (TI) for various Gd^3+^ concentrations; typical saturation trends are observed, with the magnetization recovery being faster for higher Gd^3+^ contents. (c) Longitudinal relaxation rate *R*_1_ as a function of the pH for various Gd^3+^ concentrations. (d) Longitudinal relaxation rate *R*_1_ as a function of the Gd^3+^ concentration for various pH values.

To investigate potential applications of CDs-Gd as contrast agents for MRI, *T*_1_-weighted magnetic resonance images were acquired at various Gd^3+^ concentrations and pH values by using a 1.5T clinical MRI scanner. MRI acquisitions were conducted by varying the pH to investigate the possibility of monitoring changes in the tumor microenvironment (TME) during anticancer treatments. This because the pH of the TME typically pass from acidic (pH 5.5–6.4) to physiological (pH 7.4) during the regression of cancerous diseases. Figure 5a displays *T*_1_-weighted acquisitions of CDs-Gd samples prepared at pH 5.5, 6.4, and 7.4 (presented in different rows from top to bottom) at different Gd^3+^ concentrations. The signal intensity, shown in Figure 5b, was plotted against the inversion time to determine the relaxation time *T*_1_ and the corresponding relaxation rate *R*_1_ (= 1/*T*_1_).

From Figure 5c, it can be observed that the *R*_1_ values tend to decrease as the pH value decreases. This trend is attributed to the increased concentration of H^+^ ions in the solution with lower pH, affecting proton exchange phenomena that influence longitudinal relaxation. Additionally, the hydration state of a contrast medium can alter the exchange rate of bound water, consequently modifying the MR relaxation rates.^46,47^ Therefore, it can be inferred that the pH likely affects the hydration state of CDs-Gd due to the protonation of carboxylic groups on the surface, leading to an enhanced relaxation efficiency and increased MR contrast at pH 5.5. This effect becomes more prominent with higher Gd^3+^ concentrations. The maximum variation is observed when the pH increases from 5.5 to 6.4, resulting in a maximum *R*_1_ decrease of 20% for the largest Gd^3+^ concentration of 10.8 μM. For lower Gd^3+^ concentrations, the *R*_1_ decrease with pH is smaller. The differences between pH 6.4 and 7.4 are of lesser significance or even negligible.

Figure 5d illustrates the relationship between *R*_1_ and Gd^3+^ concentrations at different pH values. A decrease in relaxation times of *T*_1_ was observed as the Gd^3+^ concentration increased, consistent with the rise in the relaxation rate *R*_1_ due to the presence of paramagnetic species. This relationship can be described by the formula *R*_1obs_ = *R*_1int_ + ρ_1_[Gd^3+^], where *R*_1obs_ represents the observed relaxation rate, *R*_1int_ is the relaxation rate in the absence of added paramagnetic species, ρ_1_ is the longitudinal relaxivity, and [Gd^3+^] is the Gd concentration.^48,49^ To determine the relaxivity of ρ_1_, the relaxation rate *R*_1_ was linearly fitted against the Gd^3+^ concentration (Figure 5d). The relaxivity values were found to be 5.0 ± 1.1 mM^–1^ s^–1^ at pH 7.4, 6.1 ± 0.7 mM^–1^ s^–1^ at pH 6.4, and 11.3 ± 0.2 mM^–1^ s^–1^ at pH 5.5. Notably, the relaxivity at pH 7.4 exceeded that of gadobutrol (3.3 ± 0.2 mM^–1^ s^–1^ in water solution), a commonly used clinical MRI contrast agent.^50^ Comparatively, the relaxivity values at pH 6.4 were approximately 20% higher, while at pH 5.5, they were about 130% higher than the reference value at pH 7.4. Furthermore, with an increase in Gd^3+^ concentration from 1.1 to 10.8 μM, a 30% increase in *R*_1_ was observed specifically at pH 5.5. Hence, the developed CDs-Gd demonstrate enhanced sensitivity to pH variations near 5.5. On the whole, CDs-Gd nanohybrids effectively distinguish between pH 5.5 and pH 6.4–7.4, thus potentially allowing TME monitoring during theranostic treatments. This opens the way to sophisticated anticancer approaches using MRI not only to stratify patients but also to personalize therapies in a noninvasive methodology.

### 3.5 Biological Characterization of the Gadolinium-Doped Carbon Nanodots (CDs-Gd)

**Figure 6:**
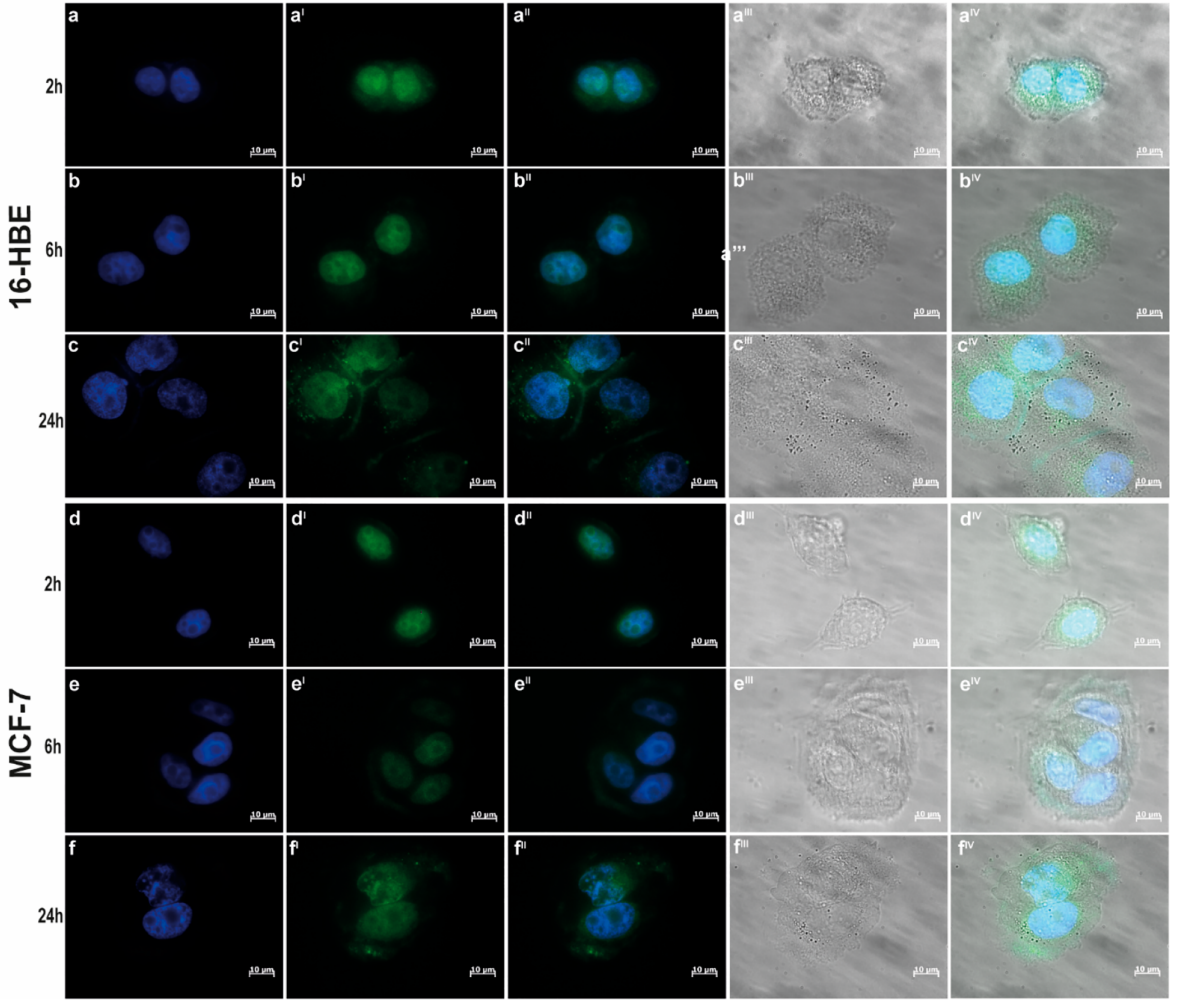
Cell uptake kinetics of CDs-Gd on breast cancer (MCF-7) and bronchial epithelial (16-HBE) cells followed by fluorescence microscopy: nuclei are stained with DAPI (a–f), CDs-Gd are self-fluorescent in green (FITC channel) (a^I^–f^I^), merge (a^II^–f^II^), brightfield micrographs (a^III^–f^III^), and all channels merged (a^IV^–f^IV^). 100× magnification.

The ability of CDs-Gd to enter cancer cells and enable in vitro and ex vivo fluorescence imaging was evaluated using both cancer cell (MCF-7) and epithelial cell (16-HBE) lines. The intrinsic brilliant photoluminescence of CDs-Gd was exploited to track cell uptake via fluorescence microscopy. In particular, cells were incubated with a CDs-Gd dispersion in DMEM (0.15 mg mL^–1^) for 2, 6, and 24 h, and then cells nuclei were stained with DAPI before the observation. As shown in Figures 6 a^I^–d^I^, CDs-Gd can enter both healthy and cancer cells after 2 h, demonstrating an excellent contrast as fluorescence imaging probes once internalized, with a bright self-luminescence in the green (FITC) channel. Besides, it is evident that the nanosystem co-localizes with DAPI, indicating a preferential accumulation in cell nuclei, as well as in the cytosol (Figures 6a^II^–a^IV^ and 6d^II^–d^IV^). Considering that CDs-Gd nanohybrids are ultrasmall 0-D nanoparticles of about 5 nm in diameter and nuclear membrane pores permit the internalization of particles up to 9 nm in diameter, the accumulation of CDs-Gd in the cell nuclei can be explained through a simple passive accumulation. The same intracellular distribution was observed after 6 h of incubation (Figures 6b–b^IV^ and 6e–e^IV^) for both cell lines studied in this work. However, after 24 h, a significant accumulation of the nanosystem within vesicular structures was revealed, which is more evident in 16-HBE (Figure 6c^I^) rather than in MCF-7 (Figure 6f^I^). This evidence suggests a possible difference in exocytosis kinetics between healthy and cancer cells, where healthy cells may exhibit earlier exocytosis of the CDs-Gd, while cancer cells retain them for a longer period of time. Similar results can be observed with lower magnification (Figure S4).

The cell uptake studies demonstrate the potential benefits of using the developed CDs-Gd for ex vivo monitoring of cancer-associated circulating cells through fluorescence imaging. At least in principle, fluorescence imaging can be used to track cells that have internalized CDs-Gd and escaped from the primary tumor by means of liquid biopsy.^51^ Moreover, the divergent time-dependent behavior of CDs-Gd following cellular internalization in healthy and cancer cells would reflect distinct cytotoxic effects upon NIR irradiation. This could potentially be helpful in selectively targeting and killing cancer cells while sparing healthy cells.

**Figure 7:**
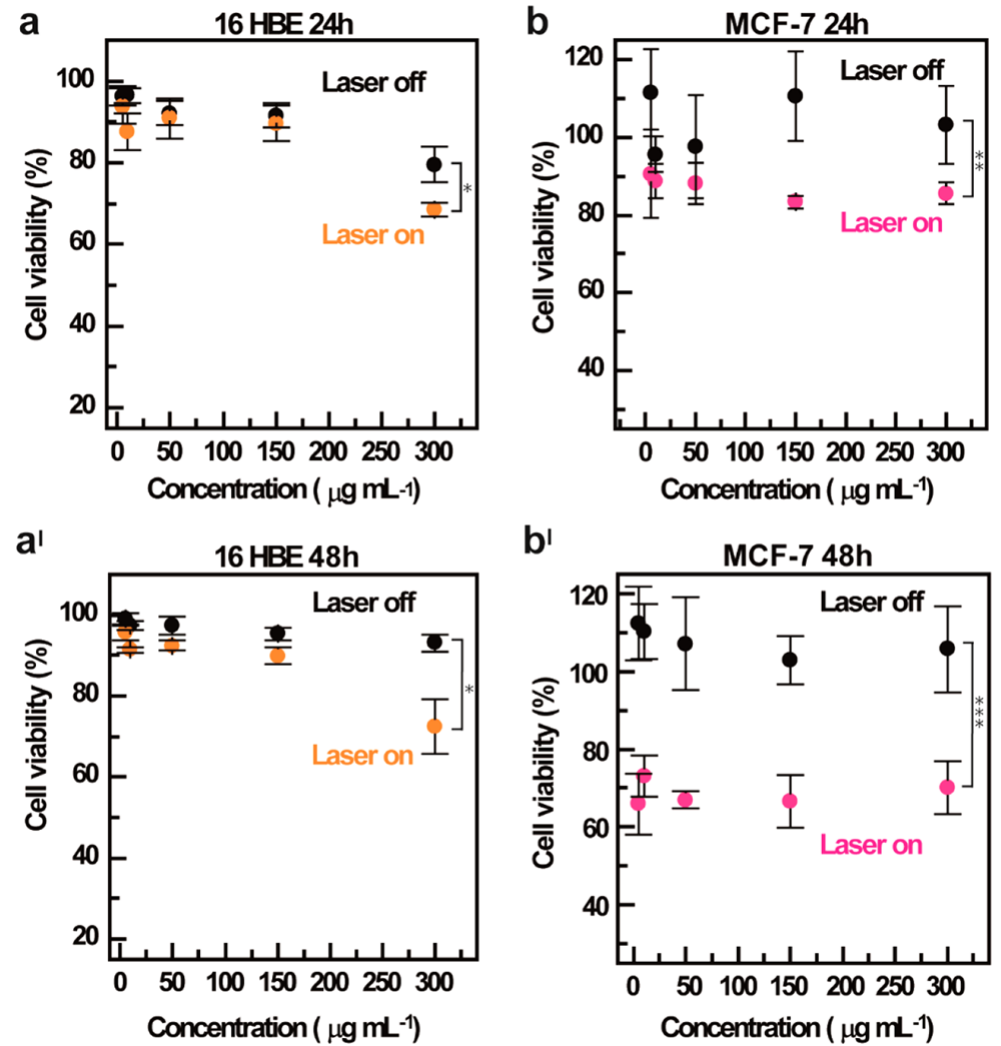
In vitro anticancer effect on breast cancer and bronchial epithelial cells. MTS assay on 16-HBE (a–a^I^) and MCF-7 (b–b^I^) after 24 h (a–b) or 48 h (a^I^–b^I^) of incubation with increasing concentrations of CDs-Gd (0.05–0.3 mg mL^–1^).

In Figure 7 we report the cell viability of MCF-7 (Figure 7a–a^I^) and 16-HBE (Figure 7b–b^I^) cells after 24 or 48 h of incubation with increasing concentrations of CDs-Gd (0.05–0.3 mg mL^–1^). It might be noticed that CDs-Gd induce negligible toxic effects on both cell lines at all the tested conditions. When incubated with CDs-Gd without external NIR light irradiation, we found that cell viability was beyond 80% at to the maximum concentration after both 24 and 48 h of incubation. On the other hand, a significant decrease in cell viability was observed for both cell lines considered upon exposure to a NIR laser beam (810 nm, 5 W cm^–2^) for 300 s. As expected, based on cell uptake evidence, photothermal-induced cytotoxic effects have a differential impact on healthy and cancer cells. Indeed, tumor cells are typically more sensitive to hyperthermia compared to healthy cells.^52,53^ Specifically, 16-HBE cells show a significant NIR-triggered reduction in cell viability only at the highest concentration tested, while the cytotoxic effect is negligible up to 0.15 mg mL^–1^. By contrast, the photothermal activation of CDs-Gd leads to efficient reduction in cancer cell viability at all the tested concentrations (Figure 7b–b^I^). After 48 h of incubation (Figure 7b^I^), the higher cytotoxic effect observed in MCF-7 cells is outstanding, indicating a time-dependent accumulation of the nanosystem in cancer cells that is consistent with the cell uptake data (Figure 6). The remarkable selectivity displayed by the NIR-triggered PTT toward breast cancer cells represents a significant advantage, especially compared with chemotherapies that indiscriminately target healthy cells, inducing the well-known adverse effects of classical anticancer treatments.

**Figure 8:**
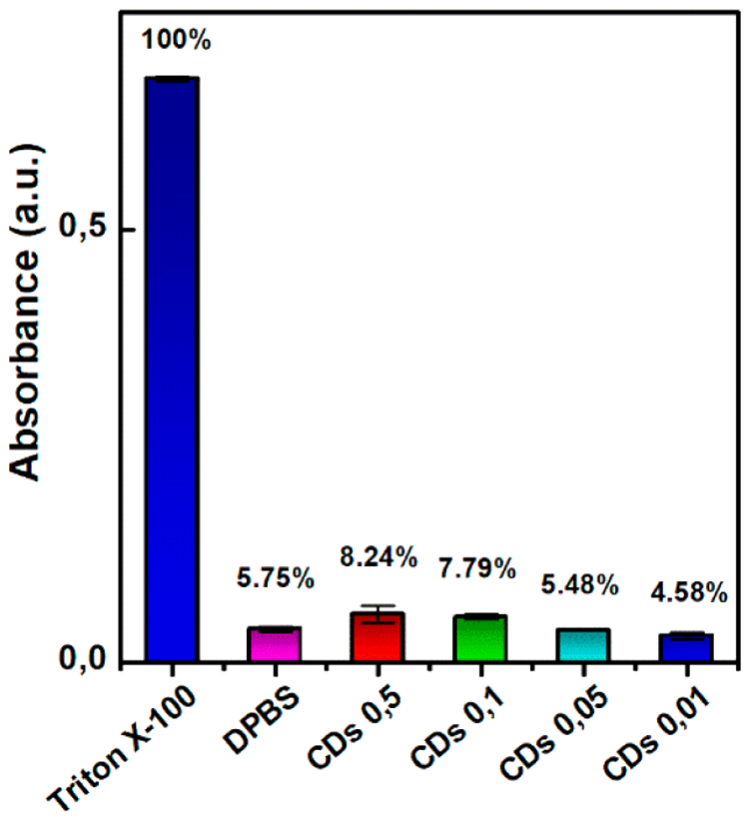
Hemolysis assay. Hemoglobin release recorded after incubation of erythrocytes (4% v/v) in DPBS pH 7.4 with increasing amount of CDs-Gd (0.01–0.5 mg mL^–1^). DPBS was used as negative control, while Triton X-100 was used as positive control.

Next, we also performed a preliminary assessment of the hemolytic activity of CDs-Gd, which must be negligible for parenteral administration of nanomedicines. We evaluated the erythrocyte lysis induced by incubation with CDs-Gd solutions (0.01–0.5 mg mL^–1^) by measuring the 520 nm absorbance of the treated solution, which is an index of hemoglobin release. The relative hemolysis rate was calculated by comparing it to the Triton X-100-induced hemolytic effects, assumed to be 100%. The results, reported in Figure 8, demonstrate that CDs-Gd do not induce a significant hemolytic effect. The highest hemolysis percentage measured was only 8.24%, which is comparable to the 5.75% hemolysis rate obtained for the negative control consisting of DPBS pH 7.4. These findings provide evidence of the excellent hemocompatibility of CDs-Gd.

## 4 Conclusions

In this work, Gd^3+^-doped carbon nanodots with homogeneous size distribution are synthesized by one-pot solvothermal decomposition of urea and Gd^3+^–citric acid complexes. We demonstrated that the Gd^3+^ doping impinges on the crystal lattice of the CDs core, providing characteristic structural defects while preserving the typical *d* spacing of graphitic cores (0.244 ± 0.013 nm). The presence of Gd^3+^ paramagnetic ions entrapped in the CDs core mitigates toxicity of free Gd^3+^ ions and does not provoke fluorescence quenching, implying significant advantages over traditional CDs. The paramagnetic component enables MRI contrast properties that are valuable for image-guided cancer therapies. The CDs core imparts fluorescence imaging features (QY = 2.1%) and NIR photothermal conversion capabilities, which allow for the ex vivo/in vitro monitoring of cancer cells and photothermal therapy (PTT) applications. Besides, we found that the relaxivity of CDs-Gd is pH-dependent (from 5.0 ± 1.1 to 11.3 ± 0.2 mM^–1^ s^–1^ at pH 7.4 and 5.5, respectively) and exceeded that of gadobutrol (3.3 ± 0.2 mM^–1^ s^–1^), enabling the MRI monitoring of pH changes in the TME that are usually observed during tumor regression. The proposed CDs-Gd nanohybrids have the ability to enter cancer cells and localize within the nuclei for extended periods of time. We also demonstrated that exposure to NIR light (810 nm, 300 s, 5 W cm^–2^) has a differential impact on healthy and cancer cells, leading to a significant decrease in cell viability preferentially in cancer cells. This implies that CDs-Gd can be remotely activated on-demand killing cancer cells with minimal local side effects. The application of this achievement is expected to develop sophisticated surface-engineered CDs hybrids for efficient and personalized multimodal image-guided anticancer approaches as well as TME sensing applications.

## Supporting Information Available

UV–vis absorption spectra of CDs-Gd fractions collected, xylenol orange assay for Gd^3+^ quantification by UV–vis spectroscopy, Gd^3+^ release kinetic, photostability study by UV–vis and fluorescence spectroscopy, cell uptake by fluorescence microscopy using a 20× magnification (PDF)
