# Supplementary material for: Gadolinium-Doped Carbon Nanodots as Potential Anticancer Tools for Multimodal Image-Guided Photothermal Therapy and Tumor Monitoring
Source: ACS Appl Nano Mater. 2023 Sep 5;6(18):17206–17. doi: 10.1021/acsanm.3c03583 (PMC10526686; doi:10.1021/acsanm.3c03583)
Supplement: Supplementary file 1 — an3c03583_si_001.pdf [file an3c03583_si_001.pdf]

Supporting Information

Gadolinium-doped Carbon Nanodots as Potential Anticancer Tools for Multimodal Image-guided Photothermal Therapy and Tumor Monitoring

Nicolò Mauro[a]*, Roberta Cillari[a], Cesare Gagliardo[b], Mara Andrea Utzeri[a], Maurizio Marrale[c,d,e], Gennara Cavallaro[a,e]

[a] Laboratory of Biocompatible Polymers, Department of "Scienze e Tecnologie Biologiche, Chimiche e Farmaceutiche" (STEBICEF), University of Palermo, Via Archirafi, 32 90123 Palermo, Italy.

[b] Department of "Biomedicina, Neuroscienze e Diagnostica Avanzata", University of Palermo, Via del Vespro 129, Palermo (Italy)

[c] Department of Physics and Chemistry "Emilio Segrè", University of Palermo, Viale delle Scienze Ed. 18 90128 Palermo, Italy

[d] National Institute for Nuclear Physics (INFN), Catania Division, Via Santa Sofia 64, 95123 Catania, Italy

[e] Advanced Technology Environment Network Center, Viale Delle Scienze Ed. 18, 90128 Palermo, Italy

*E-mail: nicolo.mauro@unipa.it; Fax: +39 09123891928; Phone: +39 09123891928

ORCID

Nicolò Mauro: 000-0003-0246-3474

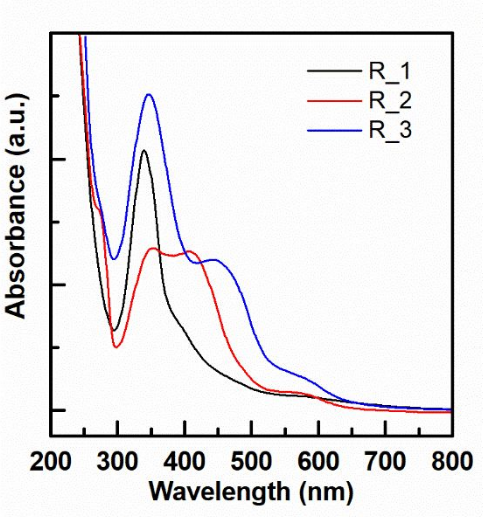

Figure S1. UV-Vis absorption spectra of fractions collected (R1→R3) after the purification of the crude CDs (R3 = CDs-Gd).

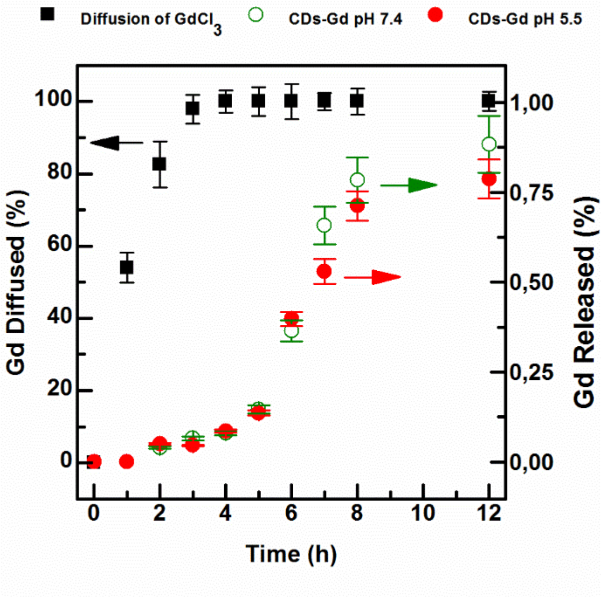

Figure S2 – Diffusion profile of $GdCl_3$ (0.25 mg mL^{-1}) and Gd^{3+} release profile at pH 5.5 (red) and 7.4 (green) from equivalent amount of purified CDs-Gd sample: the experiment was performed by equilibrium dialysis putting a solution of $GdCl_3$ (1 mL, 0.25 mg mL^{-1}) or equivalent amount of CDs-Gd in PBS pH 7.4 or acetate buffer pH 5.5 in a RC membrane with NMWCO of 500 Da, and dialyzing it against the same medium up to 12 h. The amount of Gd^{3+} ions released has been evaluated spectrophotometrically by the xylenol orange assay as reported in the main text and in Figure S3.

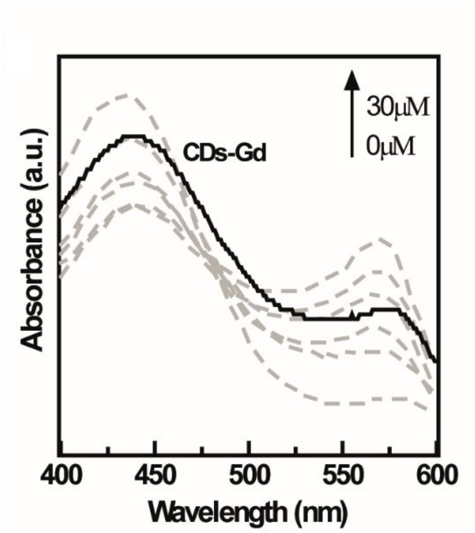

Figure S3 – Xylenol orange assay for Gd^{3+} quantification – UV-Vis absorption spectra of $GdCl_3$ standards ranging from 0 µM to 30 µM (dashed gray), and UV-Vis absorption spectrum of CDs-Gd sample (black).

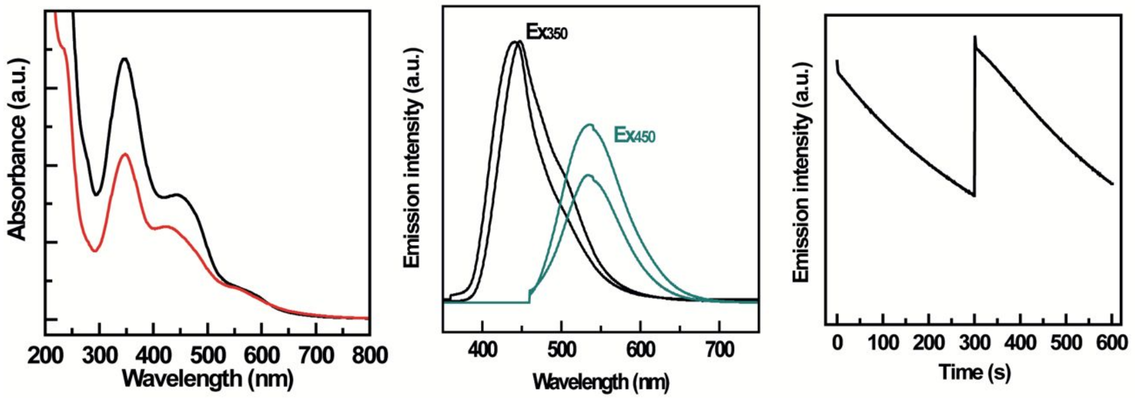

Figure S4 – Absorption (a) and emission (b) spectra of a fresh CDs-Gd dispersion in ultrapure water (0.1 mg mL^{-1}) compared with a dispersion stored for fourth month at room temperature. (c) Emission registered for a dispersion of CDs-Gd in ultrapure water (0.1 mg mL^{-1}) up to 5 minutes of continuous excitation at 350 nm (λ_{em} = 440 nm): the experiment was carried out two times with a recovery time (storage in the dark) of 10 minutes.

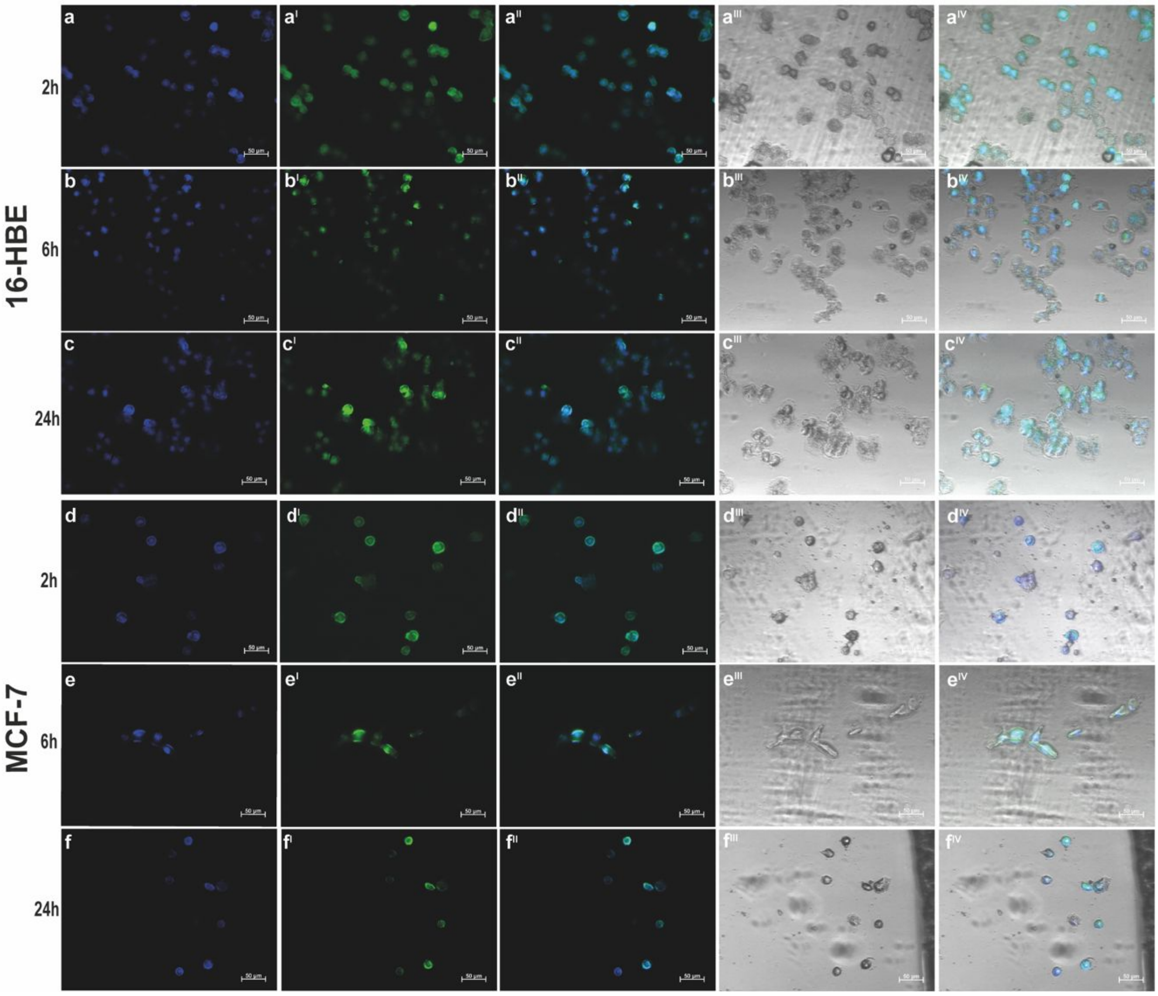

Figure S5 –Cell uptake of CDs-Gd on breast cancer (MCF-7) and bronchial epithelial (16-HBE) cells followed by fluorescence microscopy: nuclei are stained with DAPI (a-f), CDs-Gd are self-fluorescent in green (FITC channel) (a'-f'), merge (a''-f''), brightfield micrographs (a'''-f'''), and all channels merged (a'v-f'v). 20x magnification.
